# Pharmacodynamic evaluation of lefamulin in the treatment of gonorrhea using a hollow fiber infection model simulating *Neisseria gonorrhoeae* infections

**DOI:** 10.3389/fphar.2022.1035841

**Published:** 2022-11-14

**Authors:** Susanne Jacobsson, Daniel Golparian, Joakim Oxelbark, Wolfgang W. Wicha, Renata Maria Augusto da Costa, Francois Franceschi, David Brown, Arnold Louie, Steven P. Gelone, George Drusano, Magnus Unemo

**Affiliations:** ^1^ WHO Collaborating Centre for Gonorrhoea and Other STIs, National Reference Laboratory for Sexually Transmitted Infections, Department of Laboratory Medicine, Faculty of Medicine and Health, Örebro University, Örebro, Sweden; ^2^ Division of Clinical Chemistry, Department of Laboratory Medicine, Faculty of Medicine and Health, Örebro University, Örebro, Sweden; ^3^ Nabriva Therapeutics GmbH, Vienna, Austria; ^4^ Global Antibiotic Research and Development Partnership (GARDP), Geneva, Switzerland; ^5^ Institute for Therapeutic Innovation, College of Medicine, University of Florida, Orlando, FL, United States; ^6^ Nabriva Therapeutics US Inc., Fort Washington, PA, United States; ^7^ Institute for Global Health, University College London (UCL), London, United Kingdom

**Keywords:** *Neisseria gonorrhoeae*, gonorrhea, hollow fiber infection model, lefamulin, pharmacodynamics, antimicrobial treatment, pharmacokinetics

## Abstract

The emergence and spread of antimicrobial resistance in *Neisseria gonorrhoeae* is seriously threatening the treatment and control of gonorrhea globally. Novel treatment options are essential, coupled with appropriate methods to pharmacodynamically examine the efficacy and resistance emergence of these novel drugs. Herein, we used our dynamic *in vitro* hollow fiber infection model (HFIM) to evaluate protein-unbound lefamulin, a semisynthetic pleuromutilin, against *N. gonorrhoeae*. Dose–range and dose–fractionation experiments with *N. gonorrhoeae* reference strains: WHO F (susceptible to all relevant antimicrobials), WHO X (extensively drug-resistant, including ceftriaxone resistance), and WHO V (high-level azithromycin resistant, and highest gonococcal MIC of lefamulin (2 mg/l) reported), were performed to examine lefamulin gonococcal killing and resistance development during treatment. The dose–range experiments, simulating a single oral dose of lefamulin based on human plasma concentrations, indicated that ≥1.2 g, ≥2.8 g, and ≥9.6 g of lefamulin were required to eradicate WHO F, X, and V, respectively. Dose–fractionation experiments, based on human lefamulin plasma concentrations, showed that WHO X was eradicated with ≥2.8 g per day when administered as q12 h (1.4 g twice a day) and with ≥3.6 g per day when administered as q8 h (1.2 g thrice a day), both for 7 days. However, when simulating the treatment with 5–10 times higher concentrations of free lefamulin in relevant gonorrhea tissues (based on urogenital tissues in a rat model), 600 mg every 12 h for 5 days (approved oral treatment for community-acquired bacterial pneumonia) eradicated all strains, and no lefamulin resistance emerged in the successful treatment arms. In many arms failing single or multiple dose treatments for WHO X, lefamulin-resistant mutants (MIC = 2 mg/l), containing an A132V amino acid substitution in ribosomal protein L3, were selected. Nevertheless, these lefamulin-resistant mutants demonstrated an impaired biofitness. In conclusion, a clinical study is warranted to elucidate the clinical potential of lefamulin as a treatment option for uncomplicated gonorrhea (as well as several other bacterial STIs).

## Introduction

*Neisseria gonorrhoeae* is the causative agent of the sexually transmitted infection (STI) gonorrhea, and 82 million new global gonorrhea cases were estimated to occur among persons aged 15–49 years in 2020 (World Health Organization, 2021). Resistance to all antimicrobials introduced for first-line treatment of gonorrhea has emerged, threatening the management and control of gonorrhea globally. Mostly sporadic cases of resistance to the extended-spectrum cephalosporin ceftriaxone, the last remaining option for effective empiric monotherapy, are currently verified, and the level of resistance to azithromycin has increased in many countries. This development threatens the effectiveness of both ceftriaxone high-dose monotherapy and ceftriaxone plus azithromycin dual therapy (Wi et al., 2017; Day et al., 2018; Eyre et al., 2018; Unemo et al., 2019; Unemo et al., 2021; Day et al., 2022; Pleininger et al., 2022; Sánchez-Busó et al., 2022). Consequently, the WHO and the U.S. Centers for Disease Control and Prevention (CDC) have categorized *N. gonorrhoeae* as an urgent threat and a priority 2 (high) pathogen for which new antimicrobials are urgently needed (World Health Organization, 2017; Centers for Disease Control and Prevention, 2019), and companies and investors have shown interest in urgent development of new treatments and/or including *N. gonorrhoeae* in their portfolios and pipelines (Seña et al., 2020; Theuretzbacher et al., 2020).

The semisynthetic antimicrobial lefamulin is the first human systemic agent in the class of pleuromutilins. and it was approved for human use by the U.S. Food and Drug Administration (FDA) in 2019 and subsequently in other territories, including the European Union, Canada, and Taiwan. Lefamulin underwent two successful phase 3 randomized controlled clinical trials, i.e., Lefamulin Evaluation Against Pneumonia 1 and 2 (LEAP-1 and LEAP-2) (Alexander et al., 2019; File et al., 2019). Lefamulin was subsequently approved for treatment of adult community-acquired bacterial pneumonia (CABP), caused by *Streptococcus pneumoniae*, *Staphylococcus aureus*, *Haemophilus influenzae*, *Legionella pneumophila*, *Mycoplasma pneumoniae*, and *Chlamydia pneumoniae*, intravenously (150 mg every 12 h for 5–7 days) or orally (600 mg every 12 h for 5 days) (XENLETA, 2022). Safety data from phase 1, 2, and 3 clinical trials suggested that lefamulin is well-tolerated, with no reported serious adverse events (Prince et al., 2013; Alexander et al., 2019; Wicha et al., 2019a; Wicha et al., 2019b; Wicha et al., 2019c; File et al., 2019). Lefamulin has a unique mechanism of action compared to other antibiotic classes. It inhibits bacterial protein synthesis by binding selectively and specifically to the A- and P-sites of the peptidyl transferase center (PTC) in domain V of the 23s rRNA of the 50S subunit. Translation is hindered by induced fit, i.e., by tightening of the binding pocket of the bacterial ribosome around lefamulin, and correct positioning of tRNA is prevented (Eyal et al., 2016). Due to the novel target and mechanism of action, the potential for cross-resistance with other currently available antimicrobials and resistance development is considered more limited (Chahine and Sucher, 2020). Mechanisms identified to mediate resistance to pleuromutilins *in vitro* include mutations in the domain V of the 23S rRNA, including methylation of the nucleotide A2503 by the methyl transferase cfr, and mutations or deletions in the *rplC* and *rplD* genes encoding the ribosomal proteins L3 and L4, respectively. Mutations in *S. aureus* in L3 (*rplC*) at the amino acid positions 145, 148, 149, 152, 155, 157, 158, and 159 and in L4 (*rplD*) at position 68 altered and sterically hindered the correct positioning of lefamulin within the A and P sites of the PTC (Eyal et al., 2016). Furthermore, in clinical isolates of *Staphylococcus* spp. and *E. faecium* mainly isolated from animals such as swine, resistance caused by the acquisition of ATP-binding cassette F (ABC-F) proteins, such as *vga*(A–E) and *lsa*(E), which rescue the translation apparatus from ribosome-targeted antimicrobials, has been described. Finally, resistance by the acquisition of *cfr* encoding the Cfr methyltransferase has been detected, originally identified not only in coagulase-negative staphylococci (CoNS) from animals and mostly in livestock-associated staphylococci but also in a limited number of *cfr*-positive MRSA from humans (Paukner and Riedl, 2017; Mendes et al., 2019).

Lefamulin has shown potent *in vitro* activity against *N. gonorrhoeae*, i.e., with modal MIC, MIC_50_, MIC_90_, and MIC range of 0.5 mg/l, 0.25 mg/l, 1 mg/l, and 0.004–2 mg/l, respectively (Jacobsson et al., 2017). *In vitro* studies have shown promising results also for other bacterial STIs, i.e., MIC_50/90_ of 0.02/0.04 mg/l (range 0.01–0.04 mg/l) against *C. trachomatis* and MIC_50_ of 0.063 mg/l (range 0.002–0.063 mg/l) against *M. genitalium* (Jacobsson et al., 2017; Jensen and Paukner, 2017; Paukner et al., 2018). Accordingly, based on available *in vitro* data, lefamulin is promising for future treatment of gonorrhea, chlamydia and *M. genitalium* infections (Jacobsson et al., 2017; Sena et al., 2020). However, no data regarding lefamulin resistance or resistance mechanisms in *N. gonorrhoeae* (or in these other bacterial STI pathogens) or correlation between *in vitro* susceptibility, pharmacodynamics (PD), and treatment outcome have been reported. Appropriate correlations between the *in vitro* susceptibility, PD, and clinical efficacy of lefamulin are imperative to guide the optimal dosing ideal for *N. gonorrhoeae* killing and suppression of resistance emergence. For the obligate human pathogen *N. gonorrhoeae*, for which no ideal animal model exists, a dynamic *in vitro* hollow fiber infection model (HFIM), which has been stated as imperative (Seña et al., 2020; Theuretzbacher et al., 2020), was recently developed and used to simulate gonococcal infections and examine pharmacokinetic (PK)/PD parameters of zoliflodacin, enabling appropriate PD evaluations of zoliflodacin treatment of gonorrhea (Jacobsson et al., 2021; Jacobsson et al., 2022).

The present study aimed to perform a PD evaluation of lefamulin against *N. gonorrhoeae* in a dynamic *in vitro* HFIM. By performing lefamulin dose–range and dose–fractionation studies, we examined the dynamic rate of lefamulin *N. gonorrhoeae* killing and emergence of any lefamulin-resistant *N. gonorrhoeae* populations. We also evaluated the PD based on free (protein-unbound) lefamulin concentrations in human plasma and in urogenital tract tissues (main gonorrhea infection sites), which was estimated based on a rat model (Wicha et al., 2022).

## Materials and methods

### Bacterial strains

The *N. gonorrhoeae* reference strains WHO F (susceptible to all relevant antimicrobials), WHO X (extensively drug-resistant, including resistance to all extended-spectrum cephalosporins), and WHO V [high-level (MIC>256 mg/l) resistant to azithromycin and with the highest lefamulin MIC (2 mg/l)] detected in a previous study (Jacobsson et al., 2017) (Unemo et al., 2016) were examined.

### Antimicrobial susceptibility testing

For determination of lefamulin MICs (mg/l), agar dilution, according to Clinical and Laboratory Standards Institute (CLSI) guidelines (M07-A9 and M100-S24; www.clsi.org), on GCVIT agar plates (3.6% Difco GC Medium Base agar (BD, Diagnostics, Sparks, MD, United States) supplemented with 1% IsoVitalex (BD, Diagnostics)] and microbroth dilution (in triplicates) in the HFIM medium, i.e., modified Fastidious Broth (mFB) was performed, as previously described (Jacobsson et al., 2021; Jacobsson et al., 2022). Etest was used to determine MICs (mg/l) of ceftriaxone, cefixime, azithromycin, and ciprofloxacin, in accordance with the manufacturer’s instructions (bioMérieux, Marcy-l’Etoile, France), and the MICs were interpreted using breakpoints from the European Committee on Antimicrobial Susceptibility Testing (www.eucast.org/clinical_breakpoints/).

### Hollow fiber infection model

To simulate gonococcal infections and the population pharmacokinetic/pharmacodynamic (PK/PD) of therapeutic antimicrobials against *N. gonorrhoeae*, we recently developed and quality-assured a dynamic HFIM using cellulosic cartridges (FiberCell Systems Inc., Frederick, MD, United States) (Jacobsson et al., 2021; Jacobsson et al., 2022).

In brief, lefamulin was administered to the HFIM using syringe pumps, and peristaltic pumps isovolumetrically replaced lefamulin-containing broth medium with lefamulin-free medium to simulate the plasma half-life (t_1/2_) of lefamulin and free (protein-unbound fraction) lefamulin concentration–time profiles. Sampling for quantitative cultures [colony-forming units (CFUs)/ml] for total *N. gonorrhoeae* burden and possible lefamulin-resistant *N. gonorrhoeae* population and measurement of lefamulin concentrations were performed over 7 days. On the first day, 0.5 ml of *N. gonorrhoeae* cultures (18–24 h) from GCAGP agar plates (3.6% Difco GC Medium Base agar (BD, Diagnostics) supplemented with 1% hemoglobin (BD, Diagnostics), 1% IsoVitalex (BD, Diagnostics), and 10% horse serum) was inoculated in 49.5 ml of mFB and incubated at 36°C in a humidified 5% CO_2_-enriched atmosphere to mid-log phase. Then, 10 ml (∼10^5^–10^6^ CFUs/ml) of the *N. gonorrhoeae* suspension was inoculated into each HFIM cartridge to mimic a clinically relevant *N. gonorrhoeae* load (Bissessor et al., 2011; Chow et al., 2016; Priest et al., 2017; van der Veer et al., 2017). Lefamulin was administrated to mimic an adult human PK plasma concentration–time profile following a single oral dose of lefamulin (PK parameters for lefamulin 600 mg oral dose (fasted) were used: 12% fraction of free lefamulin in plasma, 9 h t_1/2_, and a 2-h infusion time, and linearly adjusted for other doses) (NAB-BC-3781-1107; ClinicalTrials.gov identifier NCT02557789). Furthermore, because the lefamulin concentration in the main STI tissues (urogenital tissues) in rats has been shown to be ≥ 5–10 times higher than the concentration in plasma (Wicha et al., 2022), 5 and 10 times the free lefamulin concentration in human plasma was also simulated. One HFIM cartridge per examined strain and experiment was used as an untreated growth control.

Dose–range experiments (*n* = 2) simulated, based on free lefamulin concentrations in human plasma, lefamulin single oral dose regimens of 0.4–1.6 g against WHO F, 0.4–3.6 g against WHO X, and 0.4–10 g against WHO V, and all experiments were followed in 7 days. Dose–fractionation experiments (*n* = 2) simulated, based on free lefamulin concentrations in human plasma, lefamulin oral daily dose of 1.2 g (approved oral treatment for CABP) and 1.5 g (because 750 mg is the highest single oral dose proven safe) against WHO F; 1.2–4.0 g against WHO X; and 1.2 and 1.5 g against WHO V; administered as one-half of the total dose given at 0 h and at 12 h (q12 h) repeatedly for 7 days, and 2.4–3.6 g against WHO X as one-third the total dose administered at 0, 8, and 16 h (q8 h) repeatedly for 7 days. Additionally, dose–fractionation experiments that simulated lefamulin oral dose regimens based on free lefamulin concentrations in urogenital tissues of rats were conducted, which have been shown to be ≥ 5–10 times higher than in plasma (Wicha et al., 2022). These dose–fractionation experiments (*n* = 2) included lefamulin daily dose of 1.2 g against WHO F and WHO X and lefamulin of 1.2 and 1.5 g for WHO V, administered as one-half of the total dose given at 0 h and at 12 h (q12 h) repeatedly for 7 days.

### Quantification of viable bacterial populations

To determine the *N. gonorrhoeae* total population and lefamulin-resistant subpopulations, the bacterial solution (1 ml) was sampled from each HFIM cartridge at time points 2, 9, 24, 48, 72, 96, 120, 144, and 168 h for the dose–range experiments; at 2, 9, 12, 14, 24, 26, 33, 36, 38, 48, 72, 96, 120, 144, and 168 h for the q12 h dose–fractionation experiments; and at 2, 8, 10, 16, 18, 24, 26, 32, 34, 40, 42, 48, 72, 96, 120, 144, and 168 h for the q8 h dose–fractionation experiments. Samples were serially diluted in mFB and quantitatively plated on GCAGP agar plates and GCAGP agar plates containing 3×MIC of lefamulin, resulting in a detection limit of ≥100 CFUs per HFIM cartridge, as previously described (Jacobsson et al., 2021; Jacobsson et al., 2022). Colony counts (log10 CFUs/ml) were quantified after incubation for up to 72 h at 36°C in a humidified 5% CO_2_-enriched atmosphere using an automated colony counter (Scan 4000, Interscience, Saint-Nom-la-Bretèche, France).

### Lefamulin concentration determination

To confirm that the predicted lefamulin PK time–concentration profiles were observed in the HFIM, broth samples (500 µl) were collected at time points 1, 2, 9, 19, 24, 48, 72, 96, 120, 144, and 168 h for the dose–range experiments; at 1, 2, 9, 12, 14, 21, 24, 26, 33, 36, 38, 45, 48, 50, 72, 74, 96, 98, 120, 122, 144, 146, and 168 h for the q12 h dose–fractionation experiments; and at 1, 2, 8, 10, 16, 18, 24, 26, 32, 34, 40, 42, 48, 50, 72, 74, 96, 98, 120, 122, 144, 146, and 168 h for the q8 h dose–fractionation experiments.

All lefamulin concentrations were determined using liquid chromatography–tandem mass spectrometry (LC-MS/MS), with instrumentation and sample preparation as previously described (Jacobsson et al., 2021; Jacobsson et al., 2022), but the internal standard was changed to piperacillin, and a mixture of methanol/acetonitrile 75/25 was used as mobile phase B. Thus, co-elution of the internal standard (piperacillin) and lefamulin was achieved.

Within-laboratory imprecision was estimated by analyzing five samples each on 5 days at three different concentrations. The coefficient of variation (CV) was calculated to 2.7% at 87 mg/L, 2.6% at 1.7 mg/L, and 4.6% at 0.035 mg/L. Linearity of response was confirmed over a range of 200–0.020 mg/L with 17 concentrations. Linear calibration was employed with weighting 1/x. Matrix effects were found to be slightly positive, on the order of 10% at 10 and 0.1 mg/L. The internal standard compensated for this effect with a slight suppression of 5% at a low level.

### Biofitness experiments

To evaluate the biofitness of the lefamulin-resistant mutant selected in the HFIM (WHO X-C395T) compared to the lefamulin-susceptible WHO X parent strain, competition experiments using co-culture were performed in the HFIM. Briefly, bacteria were harvested from GCAGP agar plates and suspended in mFB to a quantity of ∼10^5^–10^6^ CFUs/ml. Equal volumes (5 ml/strain) of the suspensions of each strain were inoculated into the same HFIM cartridge. Aliquots (1 ml) were sampled at 24, 48, 72, 96, 120, 144, and 168 h; serially diluted in mFB; and quantitatively plated on GCAGP agar plates and GCAGP agar plates containing 3×MIC of lefamulin, as previously described (Jacobsson et al., 2021; Jacobsson et al., 2022). Colony counts (log10 CFUs/ml) were quantified after incubation for up to 72 h at 36°C in a humidified 5% CO_2_-enriched atmosphere using an automated colony counter (Scan 4000, Interscience, Saint-Nom-la-Bretèche, France). The competitive index (CI; Vincent et al., 2018) was determined by dividing the ratio of the WHO X-C395T mutant to wild-type WHO X at each time point with the ratio of the WHO X-C395T mutant to wild-type WHO X in the initial inoculum.

### Population pharmacokinetic/pharmacodynamic mathematical modeling

We simultaneously modeled three system outputs for the analysis of the experimental data. The system outputs were as follows: concentration of lefamulin, total *N. gonorrhoeae* burden, and burden of *N. gonorrhoeae* with lower susceptibility/resistance to lefamulin (selected during treatment). Population modeling was performed employing the non-parametric adaptive grid (NPAG) program of Leary et al. (2001) and Neely et al. (2012). Modeling choices (such as weighting) and goodness of fit evaluations were as previously published (Brown et al., 2015). Simulation was performed with the ADAPT V Program of D’Argenio et al. (2009) using Bayesian posterior parameter estimates.

### Comparative genomic analysis

Whole-genome sequencing (WGS) was performed, as previously described (Jacobsson et al., 2016; Golparian et al., 2020a), on selected colonies from the WHO X and WHO V experiments that grew on the GCAGP agar plates containing 3×MIC of lefamulin and that were confirmed to have a lefamulin MIC of 2 mg/l (WHO X mutant) and 8 mg/l (WHO V mutant) by agar dilution. The WGS was primarily performed to identify lefamulin resistance–associated mutations. As previously described, all reads were quality-controlled and assembled using our customized CLC Genomics Workbench v20.0.4 workflow (Golparian et al., 2020b), and comparisons to the reference genomes of WHO X and WHO V (Unemo et al., 2016) were obtained within the workflow using sequence mapping and basic variant detection.

## Results

### Phenotypic and genetic characteristics of *N. gonorrhoeae* strains

The MIC of lefamulin determined by agar dilution and microbroth methods and additional relevant characteristics of the examined *N. gonorrhoeae* reference strains WHO F (susceptible to all relevant antimicrobials), WHO X (extensively drug-resistant, including resistance to all extended-spectrum cephalosporins and fluoroquinolones), and WHO V (high-level resistant to azithromycin and with the highest lefamulin MIC (2 mg/l) detected in a previous study (Jacobsson et al., 2017)) are summarized, together with additional relevant characteristics, in Table 1.

**TABLE 1:** Relevant phenotypic and genetic characteristics of *Neisseria gonorrhoeae* strains.

Strain characteristics	WHO F, Unemo et al. (2016)	WHO X, Unemo et al. (2016)	WHO V, Unemo et al. (2016)
Lefamulin agar dilution MIC (microbroth MIC)^a^	0.125 (0.125)	0.5 (0.5)	2 (1)
Ceftriaxone MIC^b^	<0.002	**2**	0.064
Cefixime MIC^b^	<0.016	**4**	<0.016
Azithromycin MIC^b^	0.125	0.5	**>256**
Ciprofloxacin MIC^b^	0.004	**>32**	**>32**
*penA*	WT	Mosaic	WT
GyrA codon S91 and D95	WT	S91F and D95N	S91F and D95G
ParC codon D86, S87, and S88	WT	S87R and S88P	S87R
*mtrR* promoter region 13 bp inverted repeat	WT	Deletion of A	Deletion of A
*mtrR* coding region	WT	WT	WT
Mosaic *mtrRCDE*	—	—	—
PorB1b codon G120 and A121	NA	G120K and A121D	G120K and A121D
16S rRNA (bp 1192)	WT	WT	WT
23S rRNA (bp 2059)	WT	WT	A2059G
23S rRNA (bp 2611)	WT	WT	WT
*rplC*	WT	WT	WT
*rplD*	WT	WT	WT
NG-MAST	ST3303	ST4220	ST8927
NG-STAR	ST2	ST226	ST225
MLST	ST10934	ST7363	ST10314

MIC, minimum inhibitory concentration; WT, wild type; NA, not applicable; bp, base pair; NG-MAST, *N. gonorrhoeae* multiantigen sequence typing; ST, sequence type; NG-STAR, *N. gonorrhoeae* sequence typing antimicrobial resistance; MLST, multi-locus sequence typing.

^a^ MIC (mg/l) was determined using agar dilution and microbroth methods for lefamulin.

^b^ MIC (mg/l) was determined using Etest (bioMérieux, Marcy-l’Etoile, France) for ceftriaxone, cefixime, ciprofloxacin, and azithromycin. Resistance, in accordance with the breakpoints from the European Committee on Antimicrobial Susceptibility Testing (www.eucast.org/clinical_breakpoints/), is indicated in bold letters.

### Hollow fiber infection model results

**FIGURE 1:**
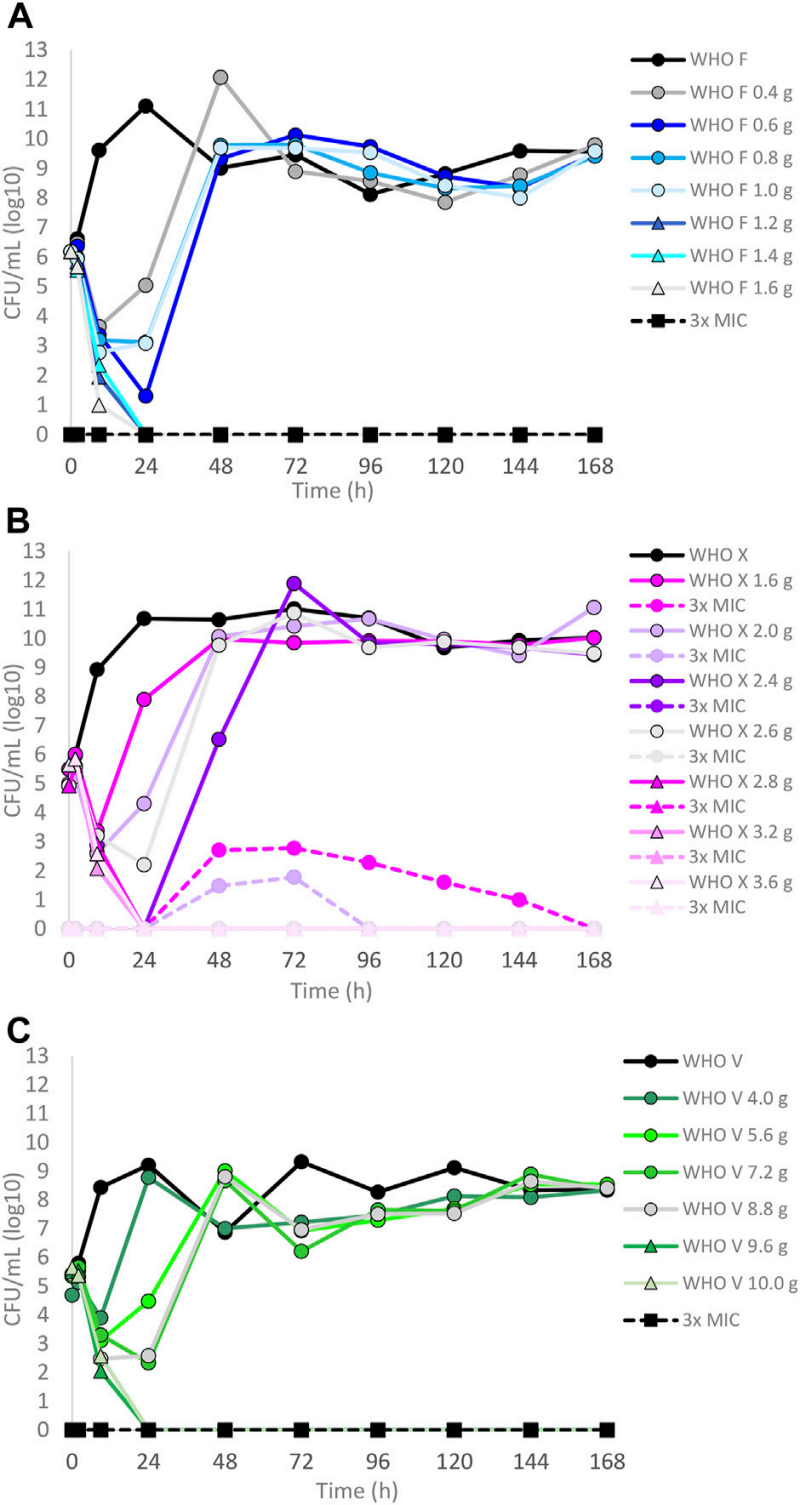
Growth curves of the total population of three *Neisseria gonorrhoeae* reference strains in the dose–range hollow fiber infection model (HFIM) experiments simulating free (12% protein-unbound) lefamulin single oral dose of **(A)** 0.4–1.6 g (WHO F), **(B)** 1.6–3.6 g (WHO X), and **(C)** 4.0–10.0 g (WHO V) and followed for 7 days. The total growth of the untreated control (black solid line) and total growth of lefamulin-resistant populations (dashed lines) on the lefamulin-containing plates (3 ×MIC) are also shown for each treatment. It is to be noted that lefamulin-resistant subpopulations only emerged for some failed treatments for WHO X.

The results of the lefamulin dose–range studies, designed based on free lefamulin concentrations in human plasma, are summarized in Figures 1A–C. Briefly, all three isolates grew well in the untreated growth control arms. At the 24-h time point, WHO X and WHO F reached a bacterial density of approximately 10^11^ CFUs/ml and WHO V around 10^9^ CFUs/ml. WHO X subsequently maintained the growth around 10^10^–10^11^ CFUs/ml throughout all 7-day experiments. WHO F and WHO V both displayed a decrease in bacterial density at the 48-h time point to 10^9^ and 10^7^ CFUs/ml, respectively, and then stabilized at approximately 10^8^–10^10^ CFUs/ml from the 72-h time point and onward (Figures 1A–C). For WHO F, a rapid *N. gonorrhoeae* kill was observed during the first 9 h with all lefamulin doses, but all treatment arms with doses lower than 1.2 g regrew after 24 h, and at 48 h and onward, the bacterial concentrations were similar to those of the untreated control arm. However, all single oral lefamulin doses of ≥1.2 g (1.2–1.6 g) eradicated WHO F*.* No lefamulin-resistant subpopulations of WHO F growing on the 3×MIC lefamulin-containing agar plates were found (Figure 1A). For WHO X, a single lefamulin dose of ≥2.8 g was needed to eradicate all bacterial growth. In the failing treatment arms with 1.0–2.2 g of lefamulin, lefamulin-resistant subpopulations were observed with *N. gonorrhoeae* colonies growing on the 3×MIC lefamulin-containing agar plates (Figure 1B). The colonies on the lefamulin-containing agar plates all displayed a lefamulin MIC = 2 mg/l using agar dilution and contained a C395T single-nucleotide polymorphism (SNP) in the *rplC* gene, which encodes an A132V amino acid substitution in ribosomal proteins L3. For WHO V, a single oral lefamulin dose of ≥9.6 g was required for eradication of all bacterial growth (Figure 1C). No lefamulin-resistant subpopulations of WHO V growing on the 3×MIC lefamulin-containing agar plates were found.

**FIGURE 2:**
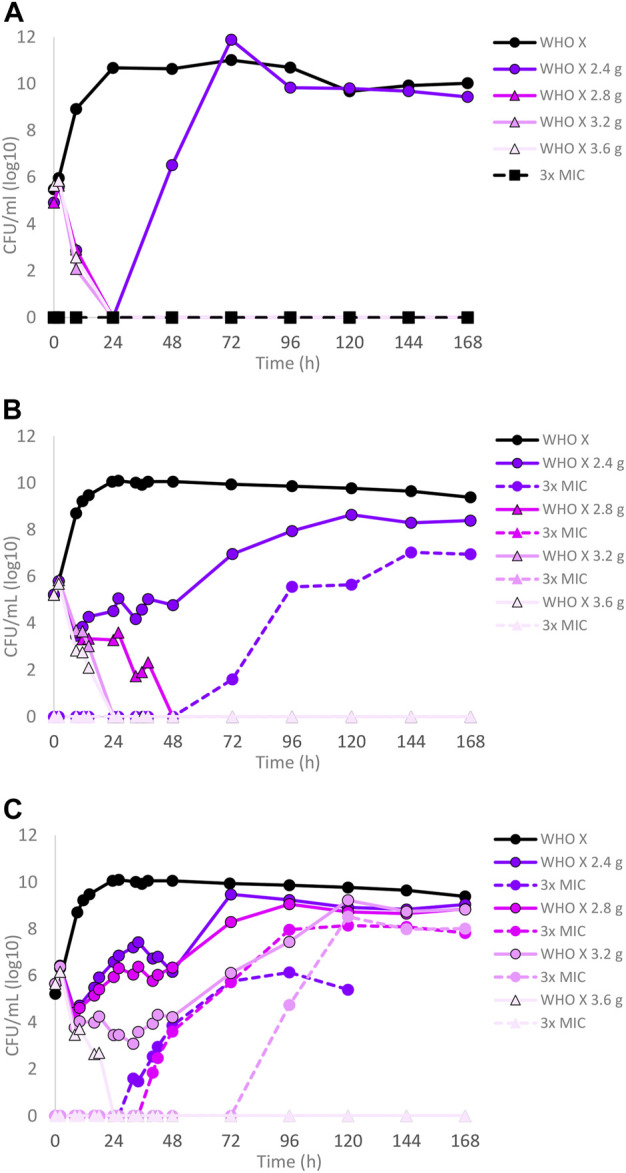
Growth curves of the total population of the *Neisseria gonorrhoeae* WHO X reference strain in the hollow fiber infection model (HFIM) experiments simulating free (12% protein-unbound) **(A)** lefamulin single oral dose of 2.4, 2.8, 3.2, and 3.6 g, **(B)** lefamulin oral daily dose of 2.4, 2.8, 3.2, and 3.6 g administered as equally divided doses q12 h for 7 days, and **(C)** lefamulin oral daily dose of 2.4, 2.8, 3.2, and 3.6 g administered as equally divided doses q8 h over 24 h for 7 days. All experiments were followed for 7 days. The total growth of the untreated control (black solid line) and total growth of lefamulin-resistant populations (dashed lines) on the lefamulin-containing plates (3 ×MIC) are also shown for each treatment.

The dose–fractionation experiments compared to the single-dose experiments for WHO X, designed based on free lefamulin concentrations in human plasma, are summarized in Figures 2A–C. Briefly, an oral lefamulin treatment with ≥2.8 g successfully eradicated WHO X growth when administered as a single dose (Figure 2A) or as ≥1.4 g of lefamulin twice a day (≥2.8 g daily dose) for 7 days (Figure 2B). However, eradication with the single-dose q24 h treatment was obtained within 24 h, while it took 48 h with the q12 h treatment (Figures 2A,B). For the q8 h treatment, ≥1.2 g of lefamulin three times a day (≥3.6 g daily dose) was needed for eradication, which was observed after 24 h (Figure 2C). As with the failing lefamulin single-dose 1.0–2.2 g q24-h treatments, lefamulin-resistant subpopulations of WHO X were observed in the failing q12-h and q8-h treatment arms, and especially the 2.4–3.2 g q8 h treatment showed substantial amplification of lefamulin-resistant subpopulations of WHO X (Figure 2C). Also, these lefamulin-resistant mutants displayed a lefamulin MIC of 2 mg/l and possessed the A132V substitution in ribosomal protein L3.

**FIGURE 3:**
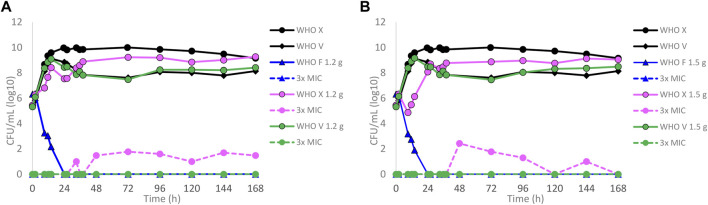
Growth curves of the total population of WHO *Neisseria gonorrhoeae* reference strains (WHO F, X, and V) in hollow fiber infection model (HFIM) experiments simulating, based on free (12% protein-unbound) lefamulin concentrations in human plasma, **(A)** lefamulin oral daily dose of 1.2 g administered as equally divided doses q12 h for 7 days and **(B)** lefamulin oral daily dose of 1.5 g administered as equally divided doses q12 h for 7 days. All experiments were followed for 7 days. The total growth of the untreated controls (black solid lines) and total growth of lefamulin-resistant populations (dashed lines) on the lefamulin-containing plates (3 ×MIC) are also shown for each treatment.

**FIGURE 4:**
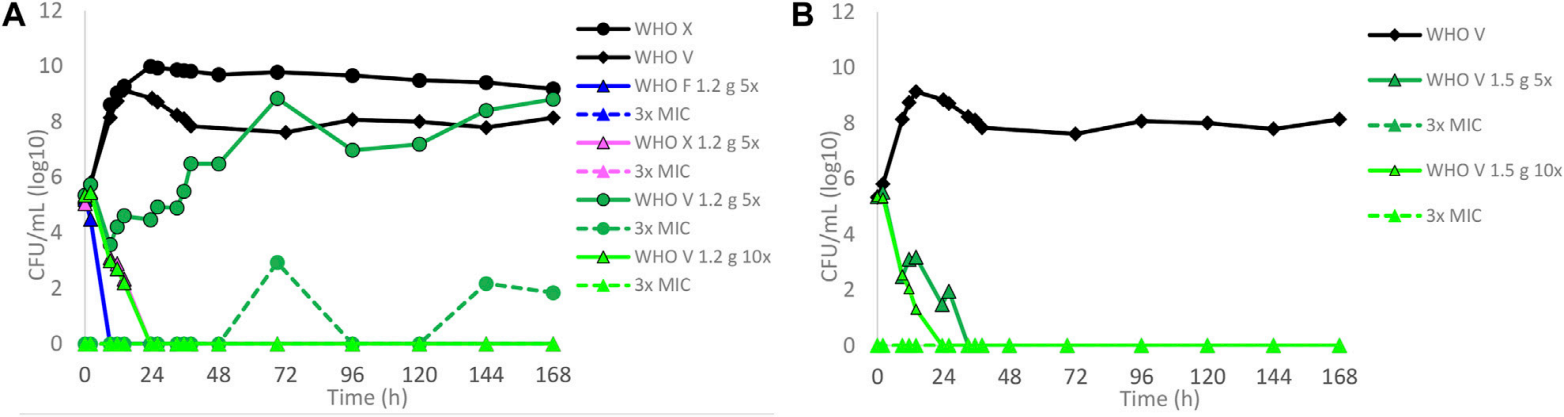
Growth curves of the total population of WHO *Neisseria gonorrhoeae* reference strains in hollow fiber infection model (HFIM) experiments simulating, based on free (12% protein-unbound) lefamulin concentrations in urogenital tract tissues (≥5–10 times higher than in plasma) that was extrapolated from a rat model (Wicha et al., 2022), **(A)** lefamulin oral daily dose of 1.2 g administered as equally divided doses q12 h for 7 days (WHO F, X, and V) and **(B)** lefamulin oral daily dose of 1.5 g administered as equally divided doses q12 h for 7 days (WHO V). All experiments were followed for 7 days. The total growth of the untreated controls (black solid lines) and total growth of lefamulin-resistant populations (dashed lines) on the lefamulin-containing plates (3 ×MIC) are also shown for each treatment.

Dose–fractionation experiments were also conducted to simulate lefamulin oral dose regimens based on free lefamulin concentration in both human plasma and urogenital tissues, which, according to a rat model, is ≥ 5–10 times higher than that in plasma (Wicha et al., 2022). In Figure 3 (based on human plasma concentrations) and Figure 4 (based on urogenital tissue concentrations), oral daily doses of lefamulin 1.2 g and 1.5 g administrated as the dose divided in two (q12 h) for 7 days for WHO F, WHO X, and WHO V are shown. The results based on free lefamulin concentrations in human plasma are shown in Figures 3A,B, and the results based on the estimated lefamulin concentrations in urogenital tissues (i.e., 5 and 10 times higher concentrations than that in plasma (Wicha et al., 2022)) are shown in Figures 4A,B. Briefly, based on human plasma lefamulin concentrations, a daily dose of 1.2 g and 1.5 g eradicated WHO F but failed to eradicate WHO X and WHO V (Figures 3A,B). However, when simulating the concentration of lefamulin to be five times higher in urogenital tissues than in plasma, a daily oral lefamulin dose of 1.2 g given as q12 h eradicated both WHO F and WHO X (Figure 4A). For WHO V, a daily dose of lefamulin 1.2 g given as q12 h and estimated to be 10 times higher in urogenital tissues than in plasma (Figure 4A) or a daily dose of lefamulin 1.5 g given as q12 h and estimated to be 5 times higher concentrations in urogenital tissues than that in plasma was required for eradication (Figure 4B). In the failing treatment arm with lefamulin 1.2 g (5×) for WHO V, sporadic, but not amplifying, lefamulin-resistant subpopulations were observed on the 3×MIC lefamulin-containing agar plates (Figure 4A). These lefamulin-resistant subpopulations had a lefamulin MIC of 8 mg/l due to the *in vitro* selected A132V substitution in ribosomal protein L3 that also emerged in the failing treatment arms for WHO X (Figures 1B, 2B,C, 3A,B).

### Population pharmacokinetic/pharmacodynamic modeling

The three-output PK/PD model was fit to all the data for WHO F and WHO X. The mean and median values for both strains are displayed in Table 2.

**TABLE 2:** Mean, median, and standard deviation of the parameter values for the hollow fiber infection model study, designed based on free lefamulin fraction in human plasma, with *Neisseria gonorrhoeae* reference strains WHO F and WHO X (in parenthesis).

Parameter	Mean	Median	Standard deviation
V_c_ (L)	4764 (4597)	4832 (4604)	368.4 (344.2)
CL (L/hr)	366.9 (289.1)	377.1 (279.8)	41.72 (29.42)
K_g-s_ (hr^−1^)	1.307 (2.335)	1.366 (2.511)	0.232 (0.329)
K_g-r_ (hr^−1^)	−(0.492)	−(0.402)	−(0.225)
K_k-s_ (hr^−1^)	2.273 (2.450)	2.081 (2.466)	0.262 (0.328)
K_k-r_ (hr^−1^)	−(1.260)	−(1.438)	−(0.539)
C_50k-s_ (mg/l)	0.066 (0.261)	0.066 (0.276)	0.023 (0.113)
C_50k-r_ (mg/l)	−(1.210)	−(0.965)	−(0.690)
H_k-s_ (---)	4.481 (8.871)	2.383 (3.466)	5.595 (7.344)
H_k-r_ (---)	−(10.95)	−(12.35)	−(7.049)
POPMAX (CFUs/ml)	3.204×10^9^ (5.149×10^10^)	7.985×10^9^ (5.049×10^10^)	8.260×10^7^ (3.551×10^10^)
IC2 (CFUs/ml)	1.920×10^6^ (3.239×10^5^)	1.847×10^6^ (3.738×10^5^)	2.900×10^5^ (1.151×10^5^)
IC3 (CFUs/ml)	−(2.770)	−(3.049)	−(1.280)

V_c_, apparent volume of the central compartment; CL, clearance; K_g-s_ and K_g-r_, rate constants of growth for the susceptible and resistant population, respectively; K_k-s_ and K_k-r_, rate constants of kill for the susceptible and resistant population, respectively; C_50k-s_ and C_50k-r_, concentrations of lefamulin at which the kill rate is half-maximal for the susceptible and resistant population, respectively; H_s_ and H_r_, Hill’s constants for the susceptible and resistant populations, respectively (unitless); POPMAX, maximal population size; CFUs, colony-forming units; IC2 and IC3, sizes of the total and resistant populations, respectively, at therapy initiation; −, no data because no lefamulin-resistant subpopulations of WHO F were growing on the 3×MIC lefamulin-containing agar plates.

The fit of the model to the data was acceptable, and the predicted–observed regressions for the analysis of WHO F and WHO X are displayed in Supplementary Figures S1, S2, respectively. The agreement between observed and predicted lefamulin concentrations in the HFIM was high (Supplementary Figures S1A,D, S2A,C, respectively).

Briefly, the mean growth rate constant for the lefamulin-susceptible populations of WHO F was only 56% of the one for WHO X; however, lefamulin-resistant subpopulations emerged only for WHO X (Table 2). Nevertheless, the mean growth rate constant for the lefamulin-susceptible populations of WHO X was about 4.8 times higher than the mean growth rate constant for the lefamulin-resistant subpopulations of this strain, which indicates a decreased biofitness of these evolved lefamulin-resistant subpopulations. The mean kill rate constants for lefamulin-susceptible populations of WHO F and WHO X were similar, and for WHO X, this constant was about twice the mean kill rate constant for the lefamulin-resistant subpopulations. Furthermore, the mean kill rate constant for the lefamulin-resistant subpopulations of WHO X was approximately 2.6 times higher than the mean growth rate constant for these lefamulin-resistant subpopulations, which further supports the biofitness disadvantage of these evolved lefamulin-resistant subpopulations. Finally, WHO F showed a more effective killing of the lefamulin-susceptible populations than WHO X, i.e., the mean kill rate constant was 1.7 times higher than the mean growth rate constant for WHO F, while the corresponding mean constants were similar for WHO X (Table 2).

### Competition biofitness experiments using coculture in the hollow fiber infection model

**FIGURE 5:**
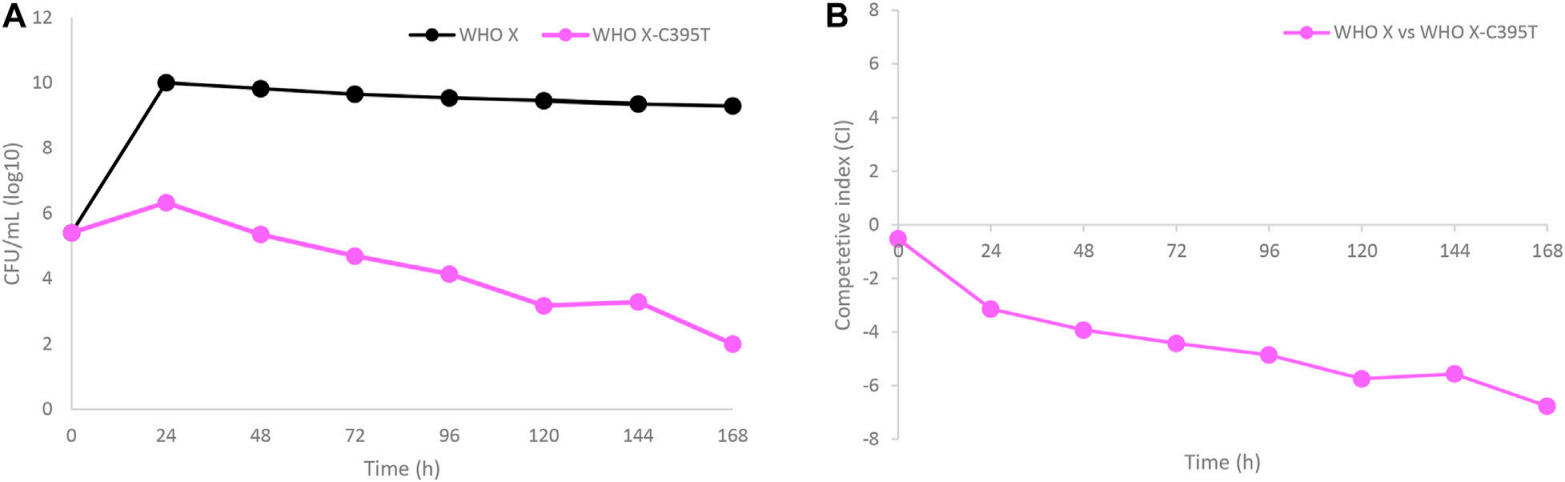
**(A)** Growth curves of the total population of the lefamulin-susceptible *Neisseria gonorrhoeae* WHO X reference strain (black line) and the *in vitro* selected lefamulin-resistant WHO X-C395T mutant (purple line), when cocultured in the same hollow fiber infection model (HFIM) cartridge and followed for 7 days. **(B)** Competitive index for the lefamulin-susceptible *Neisseria gonorrhoeae* WHO X reference strain and the outcompeted *in vitro* selected lefamulin-resistant WHO X-C395T mutant, when cocultured in the same HFIM cartridge and followed for 7 days.

WHO X and the *in vitro*-selected lefamulin-resistant WHO X-C395T mutant were co-cultured in the same HFIM cartridge for 7 days to evaluate if the *in vitro*-selected lefamulin-resistant WHO X-C395T mutant displayed any impaired bacterial growth and hence decreased biofitness (Figure 5). The growth of WHO X was maintained at approximately 10^10^ CFUs/ml during the 7-day experiment, corresponding to the same bacterial density as when monocultured. On the contrary, the growth of the lefamulin-resistant WHO X-C395T mutant was substantially lower, with only a minor initial increase to a peak at approximately 10^6^ CFUs/ml after 24 h that was followed by a steady daily decrease throughout the 7 days (Figure 5A). The calculated competitive index (Figure 5B) further confirmed that the parent WHO X strain outcompeted the lefamulin-resistant WHO X-C395T mutant, which concluded that the lefamulin-resistant WHO X-C395T mutant displayed an impaired biofitness compared to the lefamulin-susceptible WHO X parent strain.

## Discussion

Antimicrobial resistance is seriously threatening the management and control of not only gonorrhea but also other STIs, such as *M. genitalium* infections. Consequently, new treatment options for STIs are urgently needed (World Health Organization, 2017; Centers for Disease Control and Prevention, 2019; Machalek et al., 2020; Seña et al., 2020; Theuretzbacher et al., 2020; Unemo et al., 2021). Lefamulin, a novel semisynthetic pleuromutilin approved for treatment of CABP in adults (Alexander et al., 2019; File et al., 2019; XENLETA, 2022), has, in previous studies, shown a high *in vitro* activity against the etiological agents of several of the main STIs, i.e., *N. gonorrhoeae*, *C. trachomatis*, and *M. genitalium* (Jacobsson et al., 2017; Jensen and Paukner, 2017; Paukner et al., 2018). However, no data regarding the treatment of STIs with lefamulin have been published, and PK/PD data for lefamulin treatment of STIs have not been generated.

In the present study, we used our dynamic HFIM for *N. gonorrhoeae* to examine the PD of lefamulin in regard to bacterial kill and suppression of resistance development during treatment by mimicking the free lefamulin concentration–time profiles in human plasma. The dosages of drug administrated were simulated based on the concentration of free (12% protein-unbound) lefamulin in human plasma and with five and 10 times higher concentrations of free lefamulin, i.e., based on the measured ≥5–10 times higher concentrations in the urogenital tract tissues than in plasma in a rat model (Wicha et al., 2022). When simulating treatment based on free lefamulin concentrations in human plasma, successful eradication of the WHO reference strains WHO F, WHO X, and WHO V required a single oral dose of 1.2, 2.8, and 9.6 g, respectively. These results of different required lefamulin doses were in line with those of the lefamulin MICs of the three different WHO strains, where WHO V has the highest lefamulin MIC measured in a gonococcal strain (MIC = 2 mg/l) (Jacobsson et al., 2017). Giving the same total dose divided as q12 h, and not q8 h (less effective), did not improve the eradication compared to q24 h. However, by dividing the dose into q12 h, a higher total daily dose can be given. For CABP, lefamulin is licensed to be administered as 600 mg orally every 12 h for 5 days (XENLETA, 2022). When simulating this treatment and lefamulin 750 mg orally every 12 h (highest single oral dose with proven tolerability) based on free lefamulin concentrations in human plasma, only WHO F was eradicated, and both WHO X and WHO V persisted (Figure 3). Considering lefamulin concentrations in relevant STI tissues as a source control of gonococcal infections, with ≥5–10 times higher concentrations of lefamulin (Wicha et al., 2022), WHO X and WHO V were also eradicated without any lefamulin resistance emergence in the successful treatment arms (Figure 4).

The absence of human PK data from the main infection sites for gonorrhea, such as the anogenital tract and oropharynx, represents the main limitation of the present study and most similar STI studies, which is a problem not only for novel antimicrobials, such as lefamulin, but also for currently recommended antimicrobials. Simulating the treatment of STIs based on the free drug in human plasma may not always ideally reflect the anogenital and pharyngeal infection sites. Due to the lack of appropriate human PK data at these STI sites, human plasma drug concentrations are often used as a proxy, and it has been shown to frequently be valuable to answer the key questions asked in regard to PD, bacterial kill, and resistance emergence (Drusano, 2004). Nevertheless, previous PK studies have shown that lefamulin has good and rapid penetration into, for example, the interstitial space of the skeletal muscle, subcutaneous adipose tissue, and epithelial lining fluid, showing almost 6-fold higher exposure levels in the epithelial lining fluid than the free fraction in plasma already after a single intravenous dose (Zeitlinger et al., 2016). As mentioned previously, good penetration ratios of lefamulin have also been observed in other species. In a study investigating tissue penetration of lefamulin in the urogenital tract in male and female rats, the lefamulin concentrations in urogenital tissues were ≥5–10 times higher than in plasma (Wicha et al., 2022). Accordingly, we examined lefamulin treatment in our dynamic gonococcal HFIM designed based on both free lefamulin concentrations in human plasma and free lefamulin concentrations estimated in relevant tissues for gonococcal infections. For lefamulin and other STI therapeutic antimicrobials, it is imperative with improved PK data from STI-infected tissues in humans.

Previously, no data regarding lefamulin resistance in any STI agents have been reported. In the present study, we report lefamulin-resistant mutants containing a C395T SNP in the *rplC* gene, resulting in an A132V amino acid substitution in the ribosomal protein L3 (MIC≥2 mg/l) that evolved during failing lefamulin treatments, both in the dose–range and dose–fractionation experiments, especially for WHO X. Similar mutations in *rplC* have also been shown to result in lefamulin resistance in other bacterial species (Eyal et al., 2016). Fortunately, the competitive culture analysis performed indicated that the lefamulin-resistant WHO X-C395T mutant suffered from an impaired biofitness, and it was outcompeted by the lefamulin-susceptible parent WHO X strain, which indicates that these lefamulin-resistant strains may amplify less effectively, and further spread may be less likely to occur after the emergence of lefamulin resistance. Notably, inactivation of efflux pumps in *N. gonorrhoeae* was earlier investigated, and significantly decreased MICs of lefamulin, i.e., 4- to 6-fold, were shown when the MtrC-MtrD-MtrE efflux pump was inactivated. On the contrary, inactivation of the MacAB efflux pump or NorM efflux pump did not impact the activity of lefamulin (Jacobsson et al., 2017).

In conclusion, nonclinical PK/PD studies and analysis like HFIM are exceedingly valuable to support optimal dose selection decisions for novel antimicrobials and can reduce the likelihood of drug development failures and, most importantly, guide the approved dosing regimens associated with optimized patient outcomes, including complete eradication of infectious bacteria and suppression of bacterial resistance. However, the absence of human PK data from the main infection sites for STIs, such as the anogenital tract and oropharynx, is a limitation, and more appropriate human PK data from these sites are imperative to generate for novel antimicrobials, such as lefamulin, and currently recommended therapeutic antimicrobials. In the absence of lefamulin PK data in STI tissues, we examined the PD of lefamulin in the treatment of *N. gonorrhoeae* in our dynamic HFIM based on both the free lefamulin concentrations in human plasma and estimates of free lefamulin concentrations in urogenital tissues, i.e., based on PK data from a rat model (Wicha et al., 2022), which provided substantially different results. A clinical study is warranted to elucidate the true clinical potential of lefamulin as a treatment option for uncomplicated gonorrhea. It is reasonable to initially evaluate treatment with lefamulin 600 mg orally every 12 h for 5 days, which is approved for CABP (XENLETA, 2022). Finally, based on the low lefamulin MICs against *M. genitalium* and/or *C. trachomatis* (Jensen and Paukner, 2017; Paukner et al., 2018), lefamulin may be effective for treatment of several of the main bacterial STIs.

## Data Availability

The original contributions presented in the study are included in the article/Supplementary Material; further inquiries can be directed to the corresponding author.

## Supplementary material

SUPPLEMENTARY FIGURE S1
Predicted–observed regressions for lefamulin concentrations and total *Neisseria gonorrhoeae* burden for the pre-Bayesian regression (panels **A,B**) and Bayesian regressions (panels **C,D**) for WHO F.

SUPPLEMENTARY FIGURE S2
Predicted–observed regressions for lefamulin concentrations, total *Neisseria gonorrhoeae* burden, and lefamulin-resistant *N. gonorrhoeae* burden, respectively, for the pre-Bayesian regression (panels **A-C**) and Bayesian regressions (panels **D–F**) for WHO X.
