# Supplementary figures and images for: Pharmacodynamic evaluation of lefamulin in the treatment of gonorrhea using a hollow fiber infection model simulating Neisseria gonorrhoeae infections
Source: Front Pharmacol. 2022 Nov 14;13:1035841. doi: 10.3389/fphar.2022.1035841 (PMC9702083; doi:10.3389/fphar.2022.1035841)

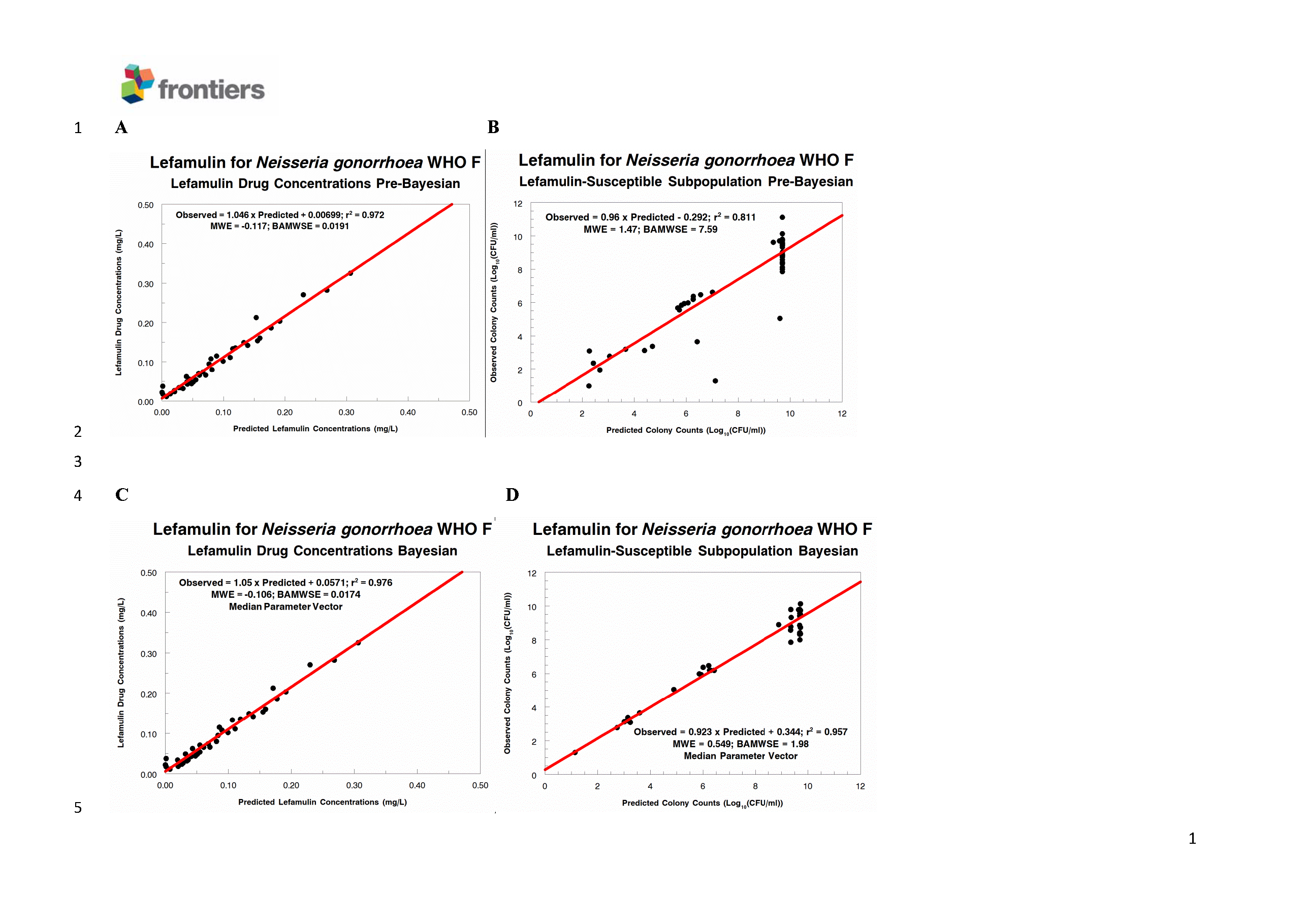

Supplement: Supplementary file 1 [file Image1.tif]

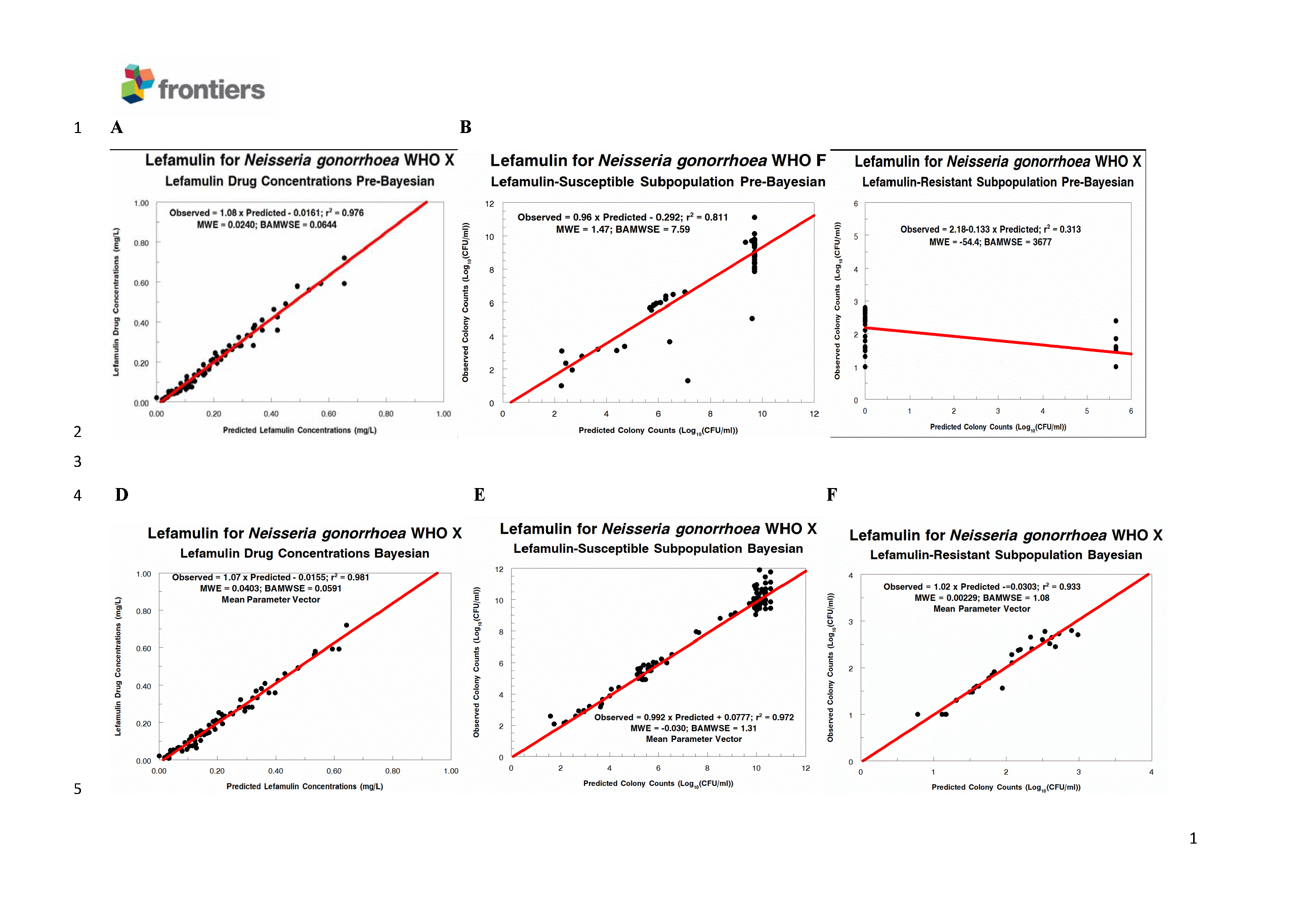

Supplement: Supplementary file 2 [file Image2.tiff]
